# Adulterated Medicine

**Published:** 1877-05

**Authors:** J. S. Pemberton

**Affiliations:** Atlanta, Ga.


### ADULTERATED MEDICINE.

BY DR. J. S. PEMBERTON, ATLANTA, GA.

We have many extraordinary accounts of the falsification of drugs and medicines, vouched for by parties best qualified to know. Instances of vile adulterations meet us every day, and it is not to be denied that there is a growing demand by a large class of dealers in drugs and medicines for cheap articles, without regard to *quality*. In England they seek to improve the quality of all remedial agents, regardless of *cost;* in this country we seek to cheapen the price of remedies regardless of quality. It is curious to compare the prices current as issued by the manufacturers and dealers in drugs and medicines, the prices varying from twice to three times as much on one list as another. The true reason and explanation of this is undoubtedly that some manufacturers and dealers make and sell preparations of full strength and known purity, while others ignore the standards of the Pharmacopœia, and sell preparations of such strength and sophisticated quality as suits themselves. We will not ask whether this is justifiable on the professional ethics, but we put it to the profession, whether it is dealing justly towards that very large class of purchasers, who are led by names, with very little knowledge as to the real merits of the preparation. Pure remedies cannot be sold for the same prices as impure ones. Those who offer medicines at reduced prices make their profits by adding often worthless articles that chemically change the action and power of remedies to such an extent that disappointment must necessarily follow their exhibition. The character of the manufacturer and dealer must secure character for his preparations. The profession should obtain a more critical knowledge of the various manufacturers and dealers, that due weight may be given to the names associated with the medicines they use, and should be careful to observe that the authenticated label of the manufacturer or druggist is affixed. The dishonest manufacturer or druggist, no less than the itinerant doctor, the quack or charlatan is an imposter, and by a system of false assurances, often obtain possession of the business which legitimately and humanely belongs to the man of science and honest purposes. The price of medicines should be a minor consideration with the physician. His aim should be to obtain pure drugs, and such cannot be obtained at the low prices quoted by some manufacturers and druggists, as the crude articles themselves actually cost more than the preparations do after they have passed through the manipulations and processes of the laboratory. Yet it is not unfrequently the case that physicians are influenced too much in their decision in favor of cheap articles, forgetting that they may not only be entirely worthless, but that the effects of the uncertainty and bad quality of these substances are transmitted directly to the practice of medicine, and in failing to fulfill the indications for their use, they not only bring distrust and discredit upon both the science and art of medicine, but also tend directly to foster and uphold the quackeries and nostrums of the day in many ways; forgetting too that perhaps one-fourth the quantity of the best preparation would answer their purpose, at a less actual cost, and at the same time would promote their reputation for success and skill. Many are the adulterations, in every conceivable manner, of remedial agents upon which the success and reputation of physicians and the life of patients depend.

The following is given as a sample of the many hundreds of articles reported, of vile adulterations. Dealers of apparent respectable standing are extensively engaged in manufacturing and selling fluid extracts, chemicals, essential oils and powdered drugs, rendering them of an entire worthless nature by adulterations. Many of the leading articles, such as sulp. quinine, ipecac., chloroform, balsam copaiba, spirits of nitre, iodide potassium, rheubarb, cream tartar, olive oil, glycerine, etc., are sold at a lower price than the crude material is worth in market. Original packages of essential oils are opened and contents adulterated and then resealed. Iodide potasium is adulterated with bromide, quinine often mixed with salacin and sulphate cinchonidia. Cream tartar is put up with tartaric acid and terra alba, regardless of the true preparation of bitartrate potas. Balsam copaiba is adulterated with linseed and other oils; spirit nitre mixed with water; glycerine reduced one-half with water; olive oil adulterated with cotton seed oil; fluid extracts are made and sold scarcely stronger than tinctures. In fact, there seems to be no limit to the villainous adulteration.

The special attention of physicians is called to the fact, and they should remember that on the purity of medicines depends numerous and important issues, such as affect his success and reputation, the interest of the sick, the mitigation and cure of diseases, the hope and expectation of anxious friends, and even in many instances, life itself. Every agent used for the mitigation or cure of disease should be given free from any adulteration. Much of a physician's success of obtaining and retaining a practice must necessarily depend upon the purity of the agents he employs. The effects of impure drugs are not limited to the physician and his confiding patient, or even his patient's friends. Communities have a great interest at stake, and the circumstances may be such that the doctor may have a public verdict against him, and his reputation, however good, however meritorious, and however hard-earned, destroyed simply by the administration of adulterated medicines. There are other issues pending, such as protracted disease and increased suffering and the loss of life, plainly to be attributed to the use of spurious medicine.

The laws of our country are framed for the punishment of frauds and its suppression, but no law has yet been passed adequate to suppress the alarming and increasing fraud—the adulteration of medicines. A counterfeit currency has laws for its prohibition, and punishment of the offending party, but alas! this is not the case with spurious drugs. And yet the man who stands convicted of making and passing counterfeit money has not committed a crime that should be mentioned in relation to its guilt and moral turpitude as contrasted with that of manufacturing and vending impure medicines. The former has aimed only to defraud in money; the latter not only takes your money, but protracts your sufferings that finally end in death. As health constitutes the crowning blessing of life, without which wealth, applause, and fame sink into absolute insignificance, so that deception which aims directly or indirectly at the citadel of health, may be in all truth said to constitute the *ne plus ultra* of fraudulent transactions, and ought to merit a general rebuke and condemnation by conscientious dealers and physicians.

Now, what is the remedy for this great evil? It is in a more liberal education and the cultivation of a higher standard of honor amongst dealers and manufacturers, and a more liberal spirit on the part of physicians in regard to prices. Let it be understood that we do not object to anyone buying things cheaply; this is in many cases the secret of success. The best remedy, to our mind, in correcting such an alarming evil, lies in a higher, broader, and more liberal education; in a close examination and scrutiny of all articles used for medical purposes, both by the druggist and physician—examinations by reagents, by physical characteristics, by the microscope, of all articles bought and offered for sale. No druggist should, and no thorough pharmaceutist will, ever place an article in his stock without a critical examination; and when adulterations are found, to condemn and report the fact through the journals should be his bounden duty.

More anon on this subject.

